# Pharmacological inhibition of USP7 suppresses growth and metastasis of melanoma cells in vitro and in vivo

**DOI:** 10.1111/jcmm.16834

**Published:** 2021-09-01

**Authors:** Minmin Xiang, Long Liang, Xinwei Kuang, Zuozhong Xie, Jing Liu, Shuang Zhao, Juan Su, Xiang Chen, Hong Liu

**Affiliations:** ^1^ Department of Dermatology Xiangya Hospital Central South University Changsha China; ^2^ Hunan Key Laboratory of Skin Cancer and Psoriasis Changsha China; ^3^ Hunan Engineering Research Center of Skin Health and Disease Changsha China; ^4^ Xiangya Clinical Research Center for Cancer Immunotherapy Central South University Changsha China; ^5^ Medical Genetics & School of Life Sciences Central South University Changsha China

**Keywords:** apoptosis, invasion, melanoma, ROS, USP7

## Abstract

Melanoma is a highly aggressive type of skin cancer. The development of diverse resistance mechanisms and severe adverse effects significantly limit the efficiency of current therapeutic approaches. Identification of the new therapeutic targets involved in the pathogenesis will benefit the development of novel therapeutic strategies. The deubiquitinase ubiquitin–specific protease‐7, a potential target for cancer treatment, is deregulated in types of cancer, but its role in melanoma is still unclear. We investigated the role and the inhibitor P22077 of ubiquitin‐specific protease‐7 in melanoma treatment. We found that ubiquitin‐specific protease‐7 was overexpressed and correlated with poor prognosis in melanoma. Further, pharmacological inhibition of ubiquitin‐specific protease‐7 by P22077 can effectively inhibit proliferation, and induce cell cycle arrest and apoptosis via ROS accumulation–induced DNA damage in melanoma cells. Inhibition of ubiquitin‐specific protease‐7 by P22077 also inhibits melanoma tumour growth in vivo. Moreover, inhibition of ubiquitin‐specific protease‐7 prevented migration and invasion of melanoma cells in vitro and in vivo by decreasing the Wnt/β‐catenin signalling pathway. Taken together, our study revealed that ubiquitin‐specific protease‐7 acted as an oncogene involved in melanoma cell proliferation and metastasis. Therefore, ubiquitin‐specific protease‐7 may serve as potential candidates for the treatment of melanoma.

## 1 INTRODUCTION

Melanoma, representing the most aggressive and the deadliest type of skin cancer, is a malignancy originating from the neural crest‐derived melanocytes in skin, uvea and mucosal tissues.^1^ The incidence rate of melanoma is lower than that of many common cancers worldwide, such as lung cancer, liver cancer and colorectal cancer, but the number of melanoma cases is increasing faster than any other types of cancer.^2^ Besides, melanoma has unusual age demography, which caused particular concern. Unlike other solid malignancies, where the majority of cases are diagnosed at the age older than 65, melanoma affects a higher proportion of younger patients, with a median age of diagnosis of 57 years.^3^ Although timely recognition, detection and appropriate surgery improved the outcomes, the prognosis of metastatic melanoma remains poor.^4^ More than 95% of melanoma patients with three or more sites of the metastatic disease die within 1 year.^5^ 75% of melanoma patients usually suffer from brain metastases that cause death in 95% of total cases.^6^ Over the past years, the inauguration of several novel systematic therapies such as BRAF‐targeted therapy and immunotherapy and advancement in local therapy have contributed to improve survival rate.^7^ Unfortunately, a large fraction of patients failed to benefit from these targeted therapies due to the skin and gastrointestinal toxicity and low efficiency. Only a minority of patients responded to the treatments.^8^ Therefore, there is an urgent need to figure out the disease pathogenesis to find new therapeutic targets and develop new drugs with low toxicity for melanoma treatment.

Ubiquitination is an important type of protein post‐translational modification (PTM), which plays a crucial role in controlling substrate degradation to ensure protein homeostasis in the cell.^9^ Deubiquitinases (DUBs) can reverse the effect of E3 ligases to remove ubiquitin from ubiquitylated proteins to regulate the stability, subcellular localization or activity of modified proteins.^10^ The role of DUBs in cancer is multifaceted, which includes proliferation, cell cycle control, cell apoptosis and DNA damage response. USP7 (ubiquitin‐specific protease‐7) is one of the deubiquitinases that belong to the UBQ‐specific protease family and plays critical roles in cancers, neurological disorders, cell differentiation, immune dysfunction, etc..^10^, ^11^ USP7 activity is highly context‐specific and exhibits its versatility in substrate selection. The multifaceted role of USP7 in various cancers is established, including lung cancer, colon cancer, breast cancer and leukaemia.^12^ Studies revealed numerous substrates of USP7 and demonstrated that the USP7 exhibits oncogenic properties. These findings make USP7 as an attractive target for pharmacological discoveries and specific treatment strategy designs.^13^ However, the role of USP7 and its therapeutic value for melanoma remain unclear.

P22077 is a specific USP7 inhibitor, which has been identified by activity‐based chemical proteomics.^14^ P22077 inhibits neuroblastoma and colon carcinoma growth via inducing p53‐mediated apoptosis.^15^ Current studies have also shown that USP7 suppressed proliferation and the colony formation capacity of lung cancer cells.^16^ However, the antitumour effect of P22077 in melanoma has not yet been studied.

We found that a high expression of USP7 in melanoma patients correlated with poor overall survival. Further, using P22077 to inhibit USP7 demonstrated a suppression in cell growth and an induction of cell cycle arrest and cell apoptosis via ROS accumulation–induced DNA damage. Moreover, P22077 also suppressed the metastasis of melanoma cells by inhibiting β‐catenin. These data indicate that USP7 inhibition could be a potential strategy for melanoma treatment.

## 2 MATERIALS AND METHODS

### 2.1 Human melanoma tissue microarray

Human melanoma tissue microarray (malignant melanoma with skin tissue array, 80 cores) was purchased from www.alenabio.com. Immunohistochemistry (IHC) staining assays using anti‐human USP7 antibody (1:1000, ab4080, abcam) were performed following standard protocols (Wuhan Servicebio Technology Co., Ltd., Hubei, China) and slides scanned using an automatic digital slide scanner (Pannoramic MIDI II). The density was quantified by using Quant software (3D Histech), and the histochemistry score (H‐score) system was used to assess the protein levels.

### 2.2 Reagents and antibodies

P22077 was purchased from Selleck (S713301). HDM2 (86934), ATM (2873), P‐ATM(13050), P‐ATR (30632), γ‐H2AX (9718), MMP9 (13667), vimentin (5741), β‐catenin (8480) and cyclin B (12231) antibodies were purchased from Cell Signaling (Cell Signaling Technology, Danvers, MA, USA). USP7(ab4080), caspase‐3 (ab13847) and cleaved‐caspase‐3 (ab214430) antibodies were purchased from abcam, and ATR (19787–1‐AP), cCdc‐2 (27334–1‐AP), P‐cCdc‐2 (21082–1‐AP) and GAPDH (60004–1‐Ig) antibodies were purchased from Protech.

### 2.3 Cell lines and cell culture

The human malignant melanoma cell lines A375 and SK‐Mel‐28 were maintained in Dulbecco's modified Eagle's medium (BI, Israel) supplemented with 10% heat‐inactivated foetal bovine serum (FBS) (BI, Israel), 100 units/ml penicillin, 100 μg/ml streptomycin and 2 mM glutamine. The mouse cell line B16F10 was maintained in RPMI 1640 containing 10% FBS, 100 units/ml penicillin, 100 μg/ml streptomycin and 2 mM glutamine. All cells were grown in a humidified incubator containing 5% CO_2_ at 37℃.

### 2.4 Cell viability assay

Cell viability assays were assessed by the Cell Counting Kit‐8 reagent (Selleck) following the manufacturer's instructions. Cells were seeded into 96‐well plates (2.5 × 10^3^ cells per well) and incubated overnight. Then, cells were cultured at various concentrations of P22077 or DMSO (control). 24, 48 and 72 h later, 10 μl of CCK‐8 was added into each well, and after 2 h of incubation, the absorbance was measured at 450 nm using the spectrophotometer (Beckman). Each experiment was performed in 6 replicates.

### 2.5 Apoptosis and cell cycle analysis

Cells were seeded into 6‐well plates (3 × 10^5^ cells per well) and incubated overnight at 37℃ in media containing 10% FBS at various concentrations of P22077 or DMSO (control). For cell cycle assays, the cells were harvested by trypsinization after 48 h. The cells were resuspended in 300 µl PBS, and subsequently, 700 µl 100% ethanol was added to the cell suspension. After incubation at 4℃.

for at least overnight, the cells were washed with PBS three times. The next day, according to the manufacturer's instructions, the collected cells were incubated at room temperature in the dark with propidium iodide (PI) (Becton Dickinson and Company). The cell cycle profile was analysed using a flow cytometer (BD Biosciences). For apoptosis assays, cells were harvested after 48 h by trypsin digestion without EDTA. The collected cells were washed with cold PBS and incubated with an Annexin V/propidium iodide stain (Becton, Dickinson and Company), according to the manufacturer's instruction. Cell apoptosis was detected by flow cytometry and analysed using FlowJo software.

### 2.6 Wound healing

5 × 10^5^ cells/well were seeded into six‐well plates and incubated under standard conditions overnight. The cells reached confluence using a 1000‐μL pipette tip to scrape the cell monolayer to create a wound. The cells were treated at various concentrations of P22077 or DMSO (control), and photomicrographs were taken at 0 h and 24 h. Representative images were captured using an inverted light microscope.

### 2.7 Transwell chamber invasion and migration assay

The Transwell chamber was placed into the corresponding culture plate. The upper chamber contains the serum‐free medium and corresponding P22077 or DMSO (control), and the lower chamber contains media containing 30% FBS. The melanoma cells were seeded in the upper chamber. After 24 h, the cells were fixed with 4% PFA and stained by 0.1% crystal violet, and migrated cell number was counted by phase‐contrast microscope and statistically analysed. To perform the invasion assay, transwell chambers were precoated with ECM Matrix gel solution (Sigma‐Aldrich) for 24 h. Residual cells on the upper transwel L chambers were counted and statistically analysed in five random fields per chamber.

### 2.8 Immunoblotting

The collected cells were treated with P22077 or DMSO for 48 h, and 200 ul of PMSF‐containing lysate was added to each sample and lysed on ice for 30 min. and The supernatant was collected by centrifugation of cells at 12000 rpm for 5 min at 4℃. Then, the BCA protein assay kit (Beyotime) was used to measure protein concentration. Proteins were detected by following the standard SDS‐PAGE and immunoblotting protocols and incubated with primary and secondary antibodies.

### 2.9 Detection of intracellular ROS generation

Cells were seeded into 6‐well plates (3 × 10^5^ cells per well) and incubated overnight at 37℃ in medium containing 10% FBS treated with P22077 or DMSO (control). The cells were processed with DCFH‐DA incubated for 20 min according to the manufacturer's instructions, and then, the intracellular fluorescence intensities were detected using a flow cytometer and analysed by FlowJo software.

### 2.10 Xenograft tumour model

1 × 10^6^ A375 cells were surgically injected into the left renal capsule of 5‐week‐old female SD nude mice. The xenografts were allowed to grow for 2 weeks before randomizing the mice into a control group and a P22077 treatment group. Animals were treated with DMSO or P22077 by intraperitoneal (i.p.) injection every day for 12 days. At the end of the experiments, all mice were killed and weighed and photographed.

### 2.11 Syngeneic lung tumour metastasis models

5 × 10^5^ B16F10 cells were injected into the tail veins of 6‐ to 8‐week‐old C57BL/6 mice maintained under specific pathogen–free conditions. Animals were treated with DMSO or P22077 by intraperitoneal (i.p.) injection every day for 16 days. At the end‐point, the mice were killed by CO_2_ asphyxiation, the lung tumours were counted, and lung images were captured.

### 2.12 Statistical analysis

Student's *t* tests and one‐ or two‐way ANOVA tests were conducted to analyse the data using the GraphPad Prism software (version 6.01). The quantified data are presented as the mean ±SEM. Differences were considered to be significant when *p* < 0.05.

## 3 RESULTS

### 3.1 USP7 is overexpressed and correlates with poor prognosis in melanoma

**FIGURE 1:**
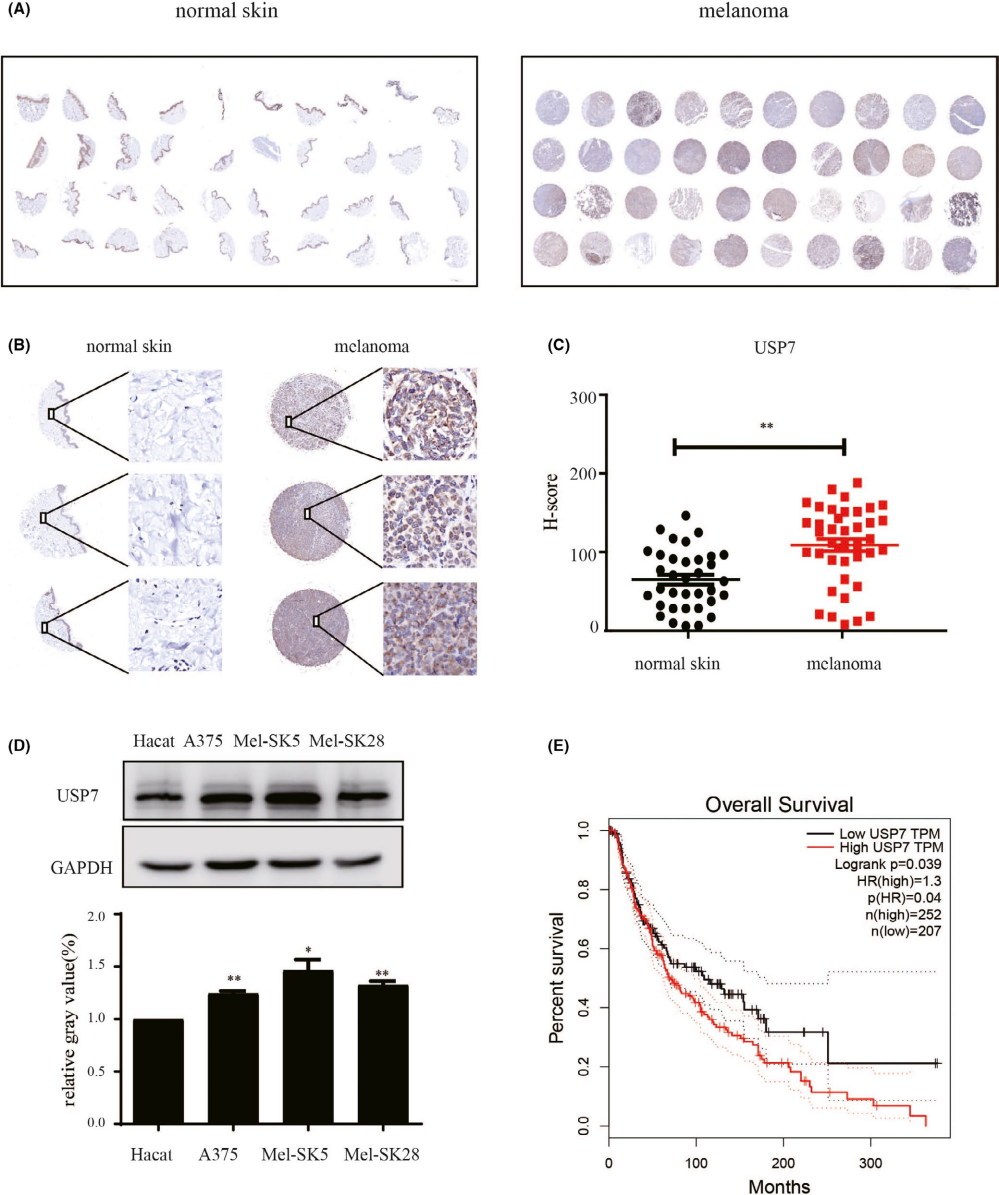
USP7 is overexpressed and correlates with poor prognosis in melanoma. Immunohistochemical staining of USP7 in normal skin and malignant melanoma. (A) The whole scanned image of the tissue array. (B) Magnified view of a tissue microarray disc with USP7 staining indicated that USP7 was highly expressed in melanoma tissues. Representative images (200× magnification). Scale bar, 50 μM. (C) The USP7 H‐score was determined by 3D Histech Quant Center and presented as dot plots (**p < 0.01). (D) The USP7 protein level was measured by Western blot in the human skin keratinocytes cell line (HaCat) and different melanoma cell lines. Quantitative analysis of USP7 protein expression compared with HaCat cell; significant differences were evaluated using Student's *t* test. *p < 0.05 vs HaCat cell. (E) Kaplan‐Meier survival analysis for the relationship between survival time of melanoma patients and the mRNA expression level of USP7 from GEPIA (cut‐off TPM 45%)

To explore the relationship between USP7 and malignancy of melanoma, first, we examined the expression level of USP7 in a human tissue microarray comprising 40 melanoma specimens from 40 patients (23 female and 17 male, age: 15–80; mean 55). The histochemical staining images were randomly selected, which showed the USP7 expression level (Figure 1A,B). The USP7 expression significantly upregulated in 77.5% of all melanomas (31/40), whereas USP7 expression was significantly downregulated in non‐malignant melanocytes (Figure 1C). Besides, compared with the normal epithelial cells, the USP7 protein level was also higher in melanoma cell lines (Figure 1D). Next, we analysed the USP7 expression in melanoma from TCGA data programmed online tool GEPIA and found that tumours with higher USP7 expression correlated with poor overall survival in melanoma (Figure 1E). Altogether, these results demonstrate that USP7 expression is upregulated in melanoma and is correlated with patients’ poor outcomes.

### 3.2 Pharmacological inhibition of USP7 by P22077 inhibits proliferation and induces cell cycle arrest and apoptosis in melanoma cells

**FIGURE 2:**
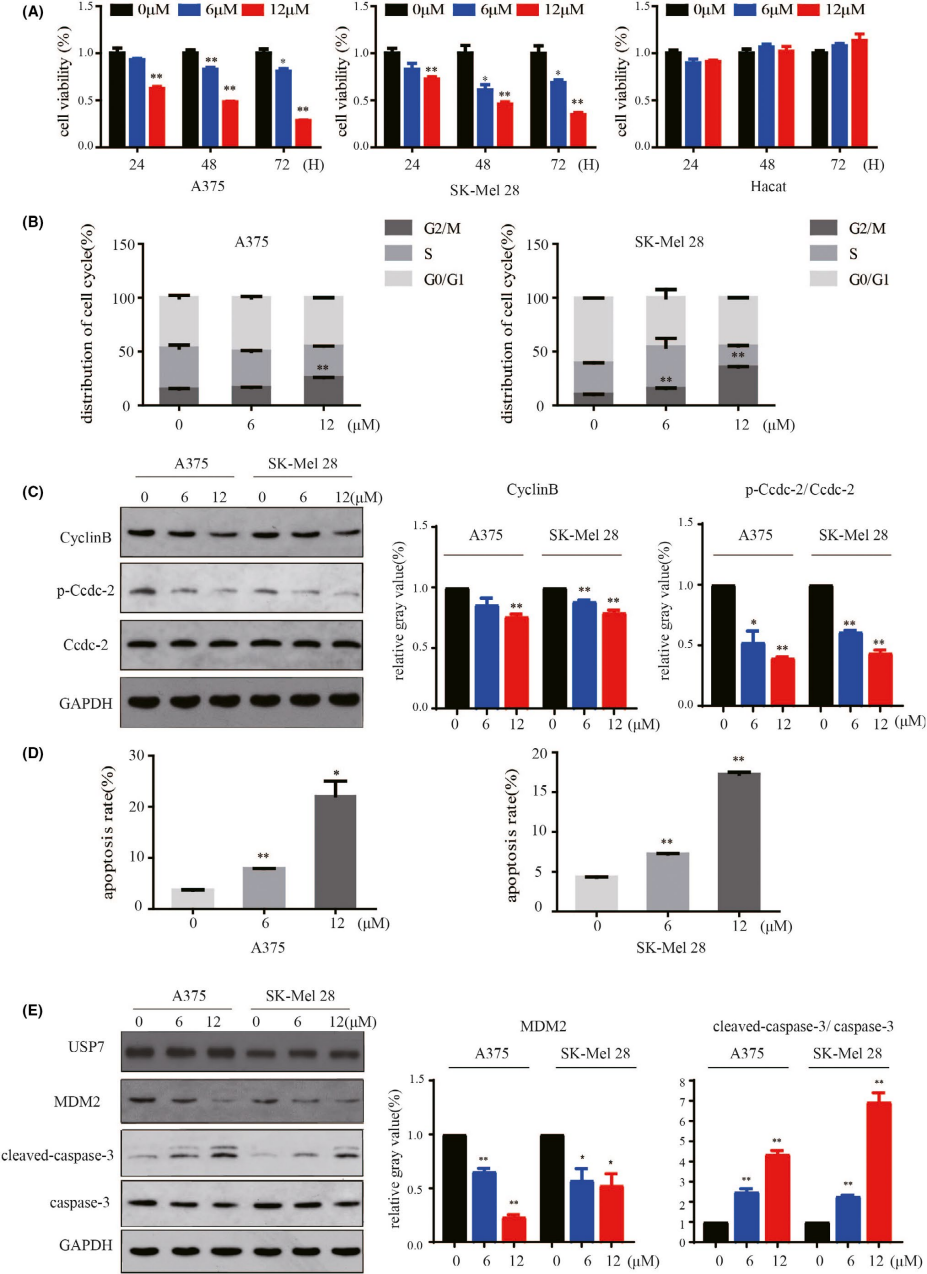
Pharmacological inhibition of USP7 by P22077 inhibits proliferation and induces cell cycle arrest and apoptosis in melanoma cells. (A) The cell viability of melanoma cell lines in the presence or absence of USP7 inhibitor P22077. Cell survival was scored in triplicate normalized to control. Values are expressed as mean and SD from three independent experiments (*p < 0.05, **p < 0.01). (B) A375 and SK‐Mel‐28 cells were treated with different dosages of P22077 for 48 h. The cell cycle distribution was detected by flow cytometry. Significant differences were evaluated using Student's *t* test. *p < 0.05 vs control. (C) Whole‐cell lysates after treatment with the indicated concentration of P22077 were subjected to immunoblotting with the indicated antibodies (Cdc2, p‐Cdc2, cyclin B) related to the cell cycle. Right panel: the results represent the mean (*n* = 3) ± SD of each group, and an asterisk (*) indicates a significant difference using Student's *t* test. *p < 0.05 vs 0 μM. (D) A375 and SK‐Mel‐28 cells were treated with various dosages of P22077 for 48 h, and the apoptosis rate was determined by flow cytometry with Annexin V and PI double staining. The results represent the mean (*n* = 3) ± SD of each group, and significant differences were evaluated using Student's *t* test. *p < 0.05 vs 0 μM. (E) Western blotting was used to analyse USP7, MDM2, caspase‐3 and cleaved‐caspase‐3 expression, which related to cell apoptosis. Right panel: the results represent the mean (*n* = 3) ± SD of each group and significant differences were evaluated using Student's *t* test. *p < 0.05 vs 0 μM

To evaluate the potential therapeutic role of USP7 inhibition in melanoma, we treated the TP53‐mutated melanoma cell line SK‐Mel‐28 and wild‐type cell line A375 with P22077. P22077 treatment significantly suppressed the proliferation of A375 and SK‐Mel‐28 cells in a dose‐dependent manner but caused no cytotoxic effect on normal human skin keratinocyte cell line (HaCat) (Figure 2A). Moreover, we found that USP7 knockdown also inhibited cell proliferation (Figure S2A,B). To investigate the effect of P22077‐induced cytotoxicity in melanoma cells, we analysed the apoptosis and cell cycle status in A375 and SK‐Mel‐28 cell lines. P22077 and USP7 knockdown also inhibited the proliferation of A375 and SK‐Mel‐28 cells by increasing the percentage of cells in G2/M and decreased the percentage of cells in G0/G1 and S phase (Figure 2B, supplement Figure S1B, supplement Figure S2C). This was confirmed by specific cell cycle protein signalling with reduced phosphorylation of cell cycle regulatory proteins Cdc2 and cyclin B (Figure 2C). We also found that USP7 inhibition and knockdown increased significantly early (Annexin+/PI−) and late apoptosis (Annexin+/PI+) regardless of P53 status (Figure 2D, supplement Figure S1A, supplement Figure S2D). Apoptosis was further confirmed by immunoblotting showing induction of the cleavage of caspase‐3. At the same time, the inhibition efficiency of P22077 was confirmed by HMD2, the known substrate of USP7. In addition, the expression of USP7 has not been affected by P22077 (Figure 2E). Together, the results suggest that USP7 inhibitor P22077 potentially induces cell cycle arrest and apoptosis in melanoma cell lines independent of the TP53 status.

### 3.3 P22077 induced DNA damage by increasing intracellular ROS level in melanoma cells

**FIGURE 3:**
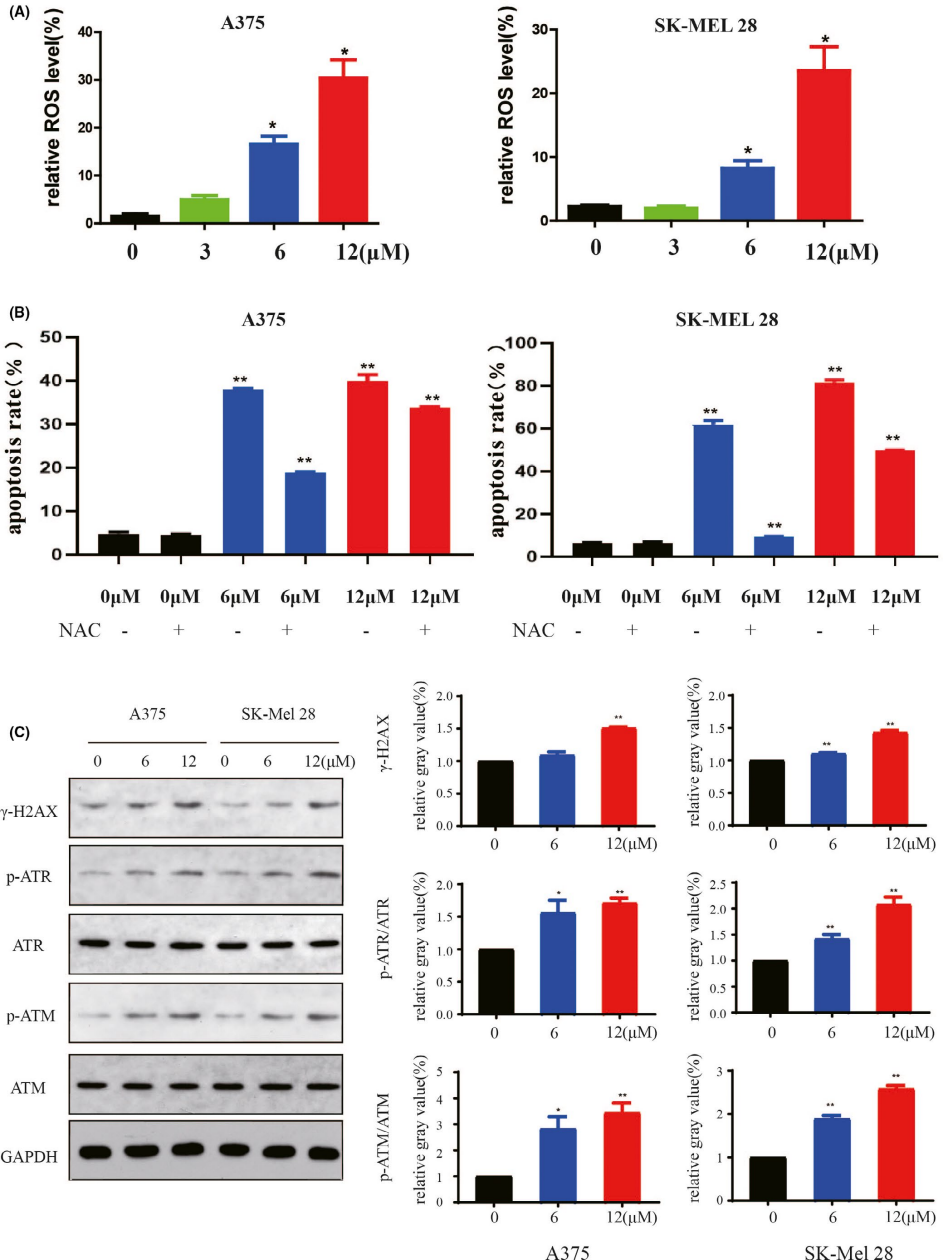
P22077 induced DNA damage by increasing the intracellular ROS level in melanoma cells. (A) A375 and SK‐Mel‐28 cells were exposed to P22077 or DMSO overnight and then treated with DCFH‐DA. The levels of ROS were measured by a DxP Athena cytometer (Cytek), and the data were analysed by the FlowJo 10 software. The results represent the mean (*n* = 3) ± SD of each group, and significant differences were evaluated using Student's *t* test. *p < 0.05 vs 0 μM (lower panel). (B) A375 and Sk‐Mel‐28 were pre‐treated with or without antioxidant NAC (2 mmol/L) for 1 h and exposed to P22077 or control medium overnight. Cells were stained with DCF fluorescence probe, and the level of ROS was measured by flow cytometry. The bar graphs showed the relative ROS levels (mean values ±SEM, *n* = 3). *p < 0.05 vs 0 μM. (C) A375 and Sk‐Mel‐28 cells were treated with P22077 for 48 h, and Western blotting was performed for the indicated antibodies (ATM, p‐ATM, ATR, p‐ATR, γH2AX) related to DNA damage. Right panel: the results represent the mean (*n* = 3) ± SD of each group, and significant differences were evaluated using Student's *t* test. *p < 0.05 vs 0 μM

At moderate levels, ROS contributes to tumour growth by acting as signalling molecules or promoting the mutation of genomic DNA, but overload ROS also prompts oxidative damage to biomacromolecules in the cell, which leads to cell dysfunction and death. Based on the previous results that P22077 treatment suppressed proliferation and induced apoptosis in melanoma cells, we hypothesized that P22077 increased ROS levels to induce melanoma cell cycle arrest and apoptosis. We applied flow cytometry to assess ROS level by intracellular DCF fluorescence and found a significant and dose‐dependent increase of intracellular levels of ROS in melanoma cell lines after an increasing concentration of P22077 treatment (Figure 3A, supplement Figure S3A,B). Furthermore, the cell apoptosis induced by P22077 could be partially blocked by the antioxidant N‐acetylcysteine (NAC), a known scavenger of ROS (Figure 3B, supplement Figure S3C). ROS generation is well known to lead to DNA damage and subsequently antitumour activity. ATM and ATR play critical roles in DNA damage responses, and these checkpoint pathways are activated by ROS‐induced DNA damage. In melanoma cell lines, P22077 treatment remarkably activated the ATM/ATR signalling pathway. The results showed that P22077 induced the upregulation of p‐ATM, p‐ATR and γH2AX expression levels (Figure 3C). These data indicate that the antimelanoma effect of P22077 is mediated through DNA damage induced by intracellular ROS.

### 3.4 P22077 significantly inhibits melanoma tumour growth in vivo

**FIGURE 4:**
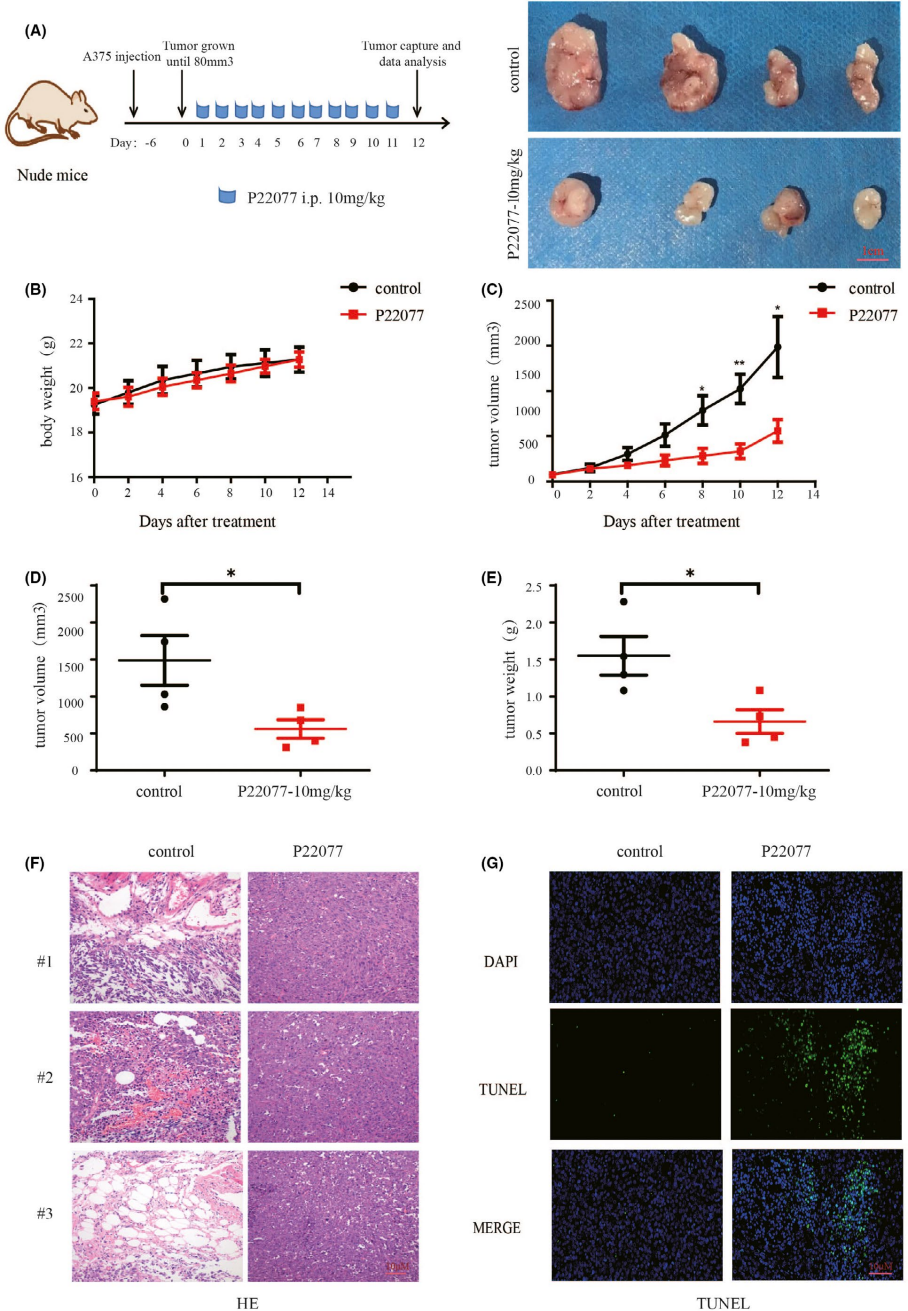
P22077 significantly inhibits melanoma tumour growth in vivo. (A) A375 melanoma cells were xenografted into nude mice. The mice were randomized for intraperitoneal injection of P22077 (10 mg/kg) or control as indicated time‐points for 12 days. Right panel: Photograph of the tumours from the mice after the mice were euthanized at the treatment end‐point. The bodyweight ((B) and tumour growth (C) were measured during the treatment period. (D) The tumour volumes in the last day. (E) Tumours were removed at the treatment end‐point, and each tumour mass from the mice was weighed (mean values ± SEM, *n* = 4). Significant differences were evaluated using a one‐way ANOVA. *p < 0.05 vs control group. Tissue sections from the tumour were fixed and stained with haematoxylin and eosin (F) and TUNEL (G) to examine the tumour histochemical morphology of apoptosis. The nucleus was stained with DAPI. Scale bar is 10 µm

Next, we investigated the in vivo antitumour effect of P22077. First, the melanoma cell line A375 was used to create a subcutaneous xenograft model in nude mice. The tumour‐bearing mice were treated with vehicle and P22077 (10 mg/kg) by intravenous (IV) injection for 12 days (Figure 4A). The results showed that P22077 treatment reduced the mouse tumour growth rate without bodyweight changes, indicating the safety of the P22077 treatment group (Figure 4B,C). Both tumour size and tumour weight were significantly decreased in the P22077‐treated group compared with the control group (Figure 4D,E). Further, the haematoxylin and eosin staining revealed a relatively much higher density of necrotic cells in P22077‐treated xenograft tumour sections compared with the control group (Figure 4F). The TdT‐mediated dUTP nick‐end labelling (TUNEL) assay showed that the percentage of apoptotic tumour cells was increased in the P22077 group compared with the vehicle group (Figure 4G). These results demonstrate the efficacy of P22077 in inhibiting tumour growth in vivo without toxicity at therapeutic doses.

### 3.5 P22077 inhibits metastasis and invasion in melanoma in vitro and in vivo

**FIGURE 5:**
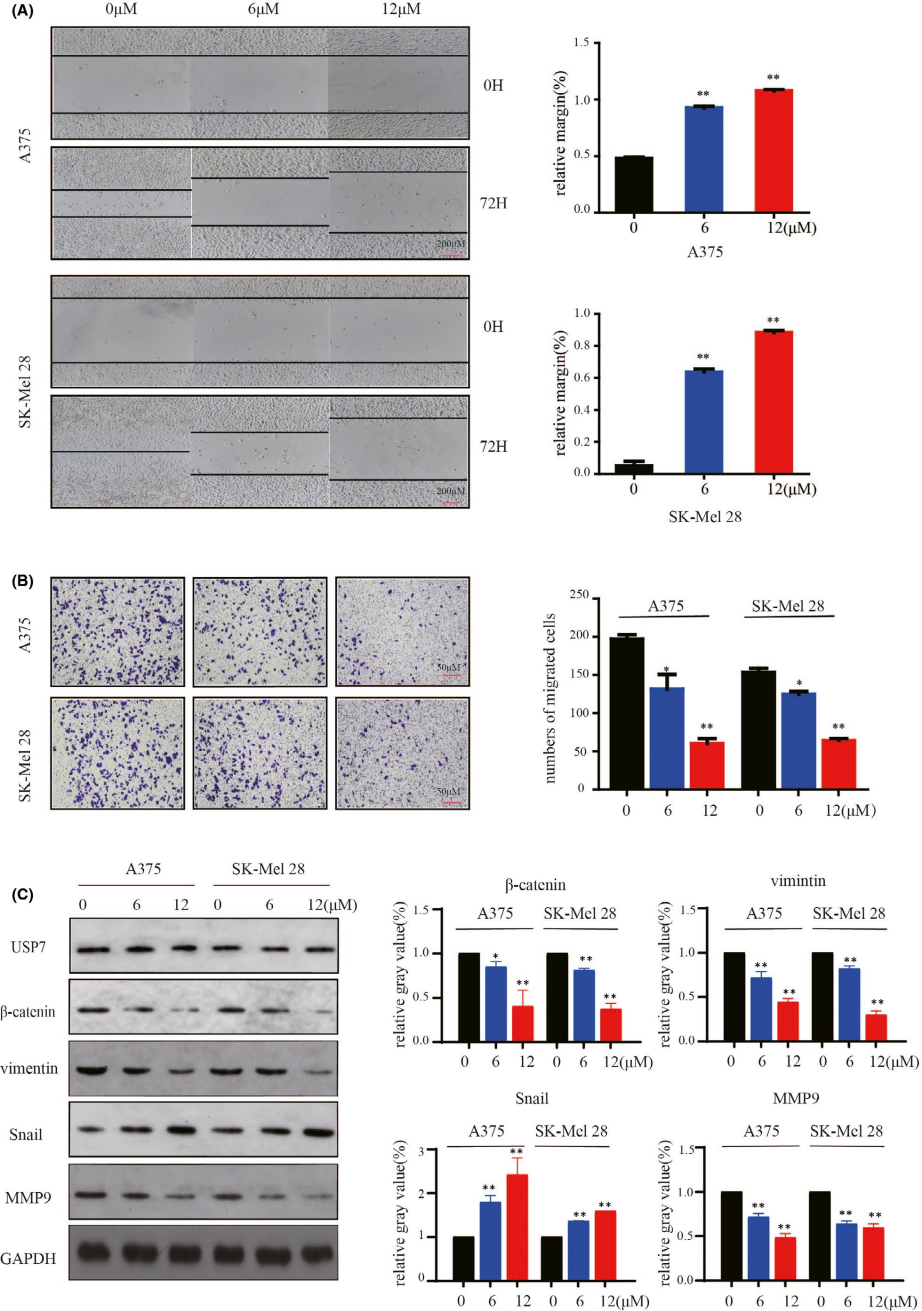
P22077 inhibits metastasis and invasion in melanoma cells. (A) A375 and SK‐Mel‐28 cells were treated at various concentrations of P22077 or DMSO. Then, the plates were scratch‐wounded with a 200‐μl sterile pipette tip and then incubated. The healing was recorded with a phase‐contrast microscope after 72 h. Right panel: quantitative analysis of relative margin(%) (mean values ± SEM, *n* = 3). Significant differences were evaluated using a one‐way ANOVA. *p < 0.05 vs control. (B) Transwell invasion assay determination of the number of melanoma cells that crossed the Matrigel layer after being treated with P22077 or DMSO (control). Right panel: the results represent the mean (*n* = 3) ± SD of each group, and significant differences were evaluated using Student's *t* test. *p < 0.05 vs 0 μM. (C) A375 and Sk‐Mel‐28 cells were treated with P22077 for 48 h, and Western blotting analysis of the expression of USP7 and some metastasis markers (β‐catenin, vimentin, snail, MMP9). Right panel: the results represent the mean (*n * =  3) ± SD of each group, and significant differences were evaluated using Student's *t* test. *p < 0.05 vs 0 μM

**FIGURE 6:**
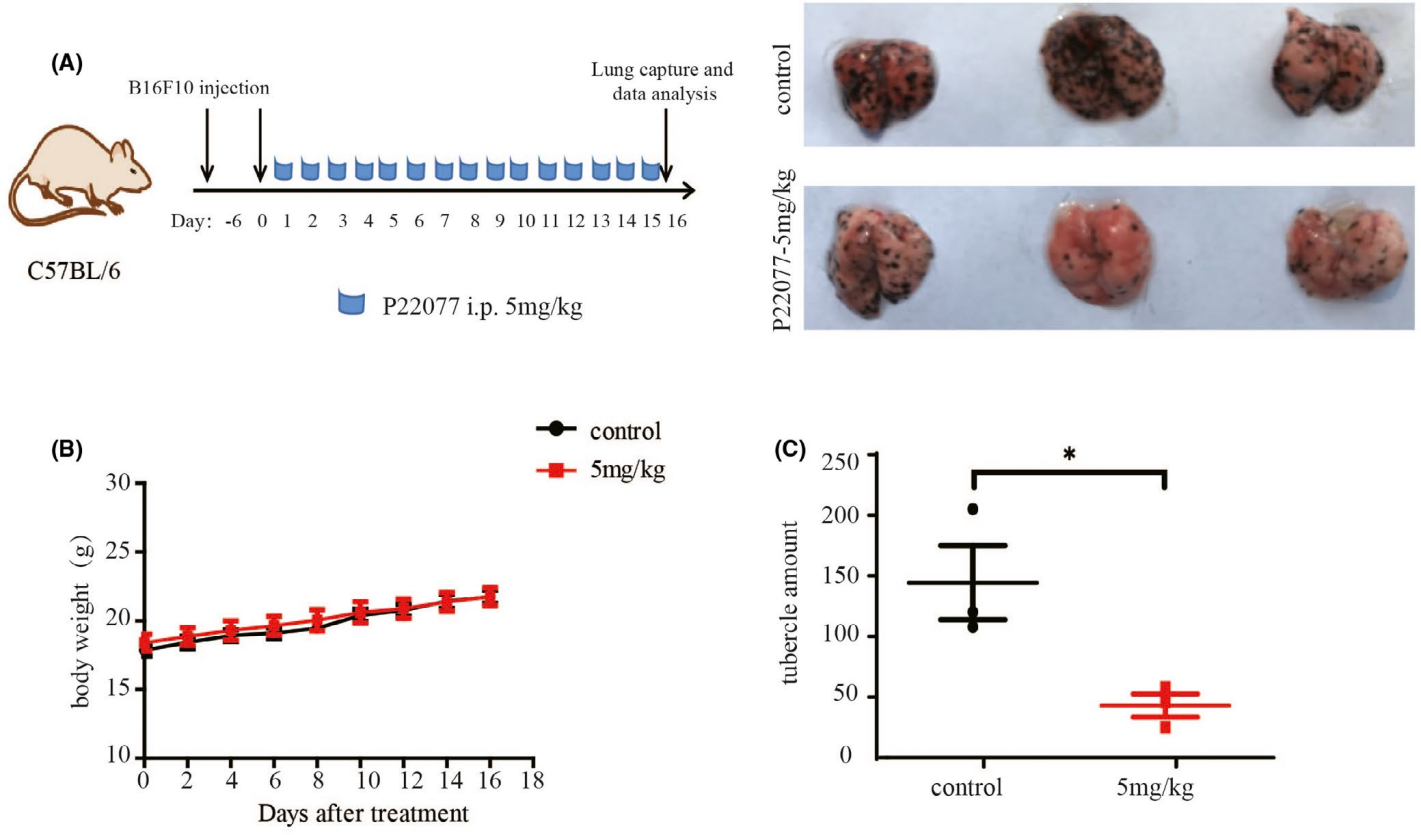
P22077 inhibits metastasis and invasion in vivo. (A) Melanoma cells from mice B16F10 were intramuscularly injected into C57BL/6 mice. The tumour‐bearing mice were randomized for intraperitoneal injection of P22077(5 mg/kg) or control as indicated time‐points for 16 days. Representative photographs of Bouin's fixed lung specimens of mice injected with P22077 or vehicle. (B) The bodyweight was measured during the treatment period. (C) The nodules in lung tissue were counted and recorded. The results were shown as the mean tumour volume ±SD, and significant differences were evaluated using a one‐way ANOVA. *p < 0.05 vs control

The epithelial‐mesenchymal transition (EMT) is a key process for promoting tumour cell invasion and metastasis.^17^ Based on the aforementioned results, we evaluated the effect of P22077 on the capability of migration by performing a wound‐healing assay. The results revealed a decreased closure of the wound area compared with control cells after P22077 treatment and knockdown (Figure 5A, supplement Figure 2E). Next, the effect of USP7 inhibition on the invasive ability of cells was accessed by transwell assays. P22077 and USP7 knockdown showed a dramatic attenuation of the invasive capacities of A375 and SK‐Mel‐28 cells compared with untreated cells (Figure 5B, supplement Figure 2F). To further explore the underlying molecular mechanism of the P22077 effect on the EMT, the EMT‐associated protein markers were examined after P22077 treatment. The results demonstrated that the inhibition of USP7 significantly induced the expression of the epithelial marker snail and reduced the mesenchymal markers vimentin and MMP9 significantly (Figure 5C). The expression of β‐catenin, a key nuclear effector of canonical Wnt signalling, was also downregulated, which implied that P22077 could inactivate the Wnt/β‐catenin pathway to inhibit EMT (Figure 5C). To further demonstrate the effect of P22077 on melanoma metastasis, the B16F10 cells were injected through the lateral tail vein to induce melanoma lung metastasis in C57BL/6 mice. The results showed that after P22077 treatment, the lung metastases were decreased significantly and without toxicity (Figure 6A‐C). These findings suggest that P22077 exerts antimetastatic activity in vitro and in vivo.

## 4 DISCUSSION

Melanoma is one of the most malignant diseases worldwide, the most aggressive and deadliest type of skin cancer.^18^ Uncontrolled tumour growth and distant metastasis remain to be huge obstacles to effective treatments. Although the favourable results were obtained from immunotherapy, in general, only a small subset of patients showed a promising response. To identify novel targets that contribute to the growth and metastasis of melanoma to develop innovative strategies is still needed. In this study, we found that a notable number of melanoma patients contain high USP7 expression, which correlates with reduced overall survival. Pharmacological inhibition of USP7 by P22077 reduces melanoma cell proliferation and induces cell cycle arrest and apoptosis independent of the TP53 status. The antimelanoma activity of P22077 might be mediated through induced intracellular ROS leading to DNA damage and subsequent cell death. P22077 is also active without toxicity at a therapeutic dose against melanoma xenograft and inhibits invasion and metastasis of melanoma cells in vitro and in vivo. Our data suggest that targeting USP7 would provide a potential strategy for melanoma treatment.

USP7 is the most widely studied deubiquitinating enzyme with numerous substrates including viral proteins, transcription factors and epigenetic modulators.^19^ In a wide variety of cancers, USP7 acts as an oncogene a good therapeutic target for cancer, and the high expression of USP7 leads to the progression of cancer.^20^ However, USP7 plays a variety of roles in tumours through the stabilization of different substrates. For example, the tumour suppressor protein p53 was the first identified deubiquitination substrate for USP7, this attributed to USP7 as a tumour suppressor, given its ability to increase the stability of p53, resulting in repression of cancer cell growth and activation of apoptotic pathways.^21^ A later study showed the p53 E3 ligase mouse double minute 2 homolog (MDM2) is also regulated by USP7‐mediated deubiquitination, leading to the degradation of p53 and reversal of the above‐mentioned cellular phenotype.^22^ Though, in normal conditions without DNA damage induction, the USP7 has a higher affinity with MDM2.^23^ Our data showed an increase in USP7 levels in melanoma cells independent of TP53 status, which indicated that USP7 may be active through other substrates to act as an oncogene in melanoma. Previous studies also have shown that USP7 regulates serval substrates to promote carcinogenesis independent of p53. These include controlling subcellular localization of PTEN to promote AML progression, stabilization of the histone demethylase PHF8 to promote breast carcinogenesis, and stabilization of N‐Myc to promote neuroblastoma progression, among others.^24^, ^25^, ^26^ Though we found an oncogenic role of USP7 with melanoma, nonetheless, the substrate requirement for USP7 to promote the progression in melanoma required further exploration.

USP7 is a promising anticancer therapeutic target because of its aberrant expression and the oncogenic role in various cancers. There are various small‐molecular inhibitors such as HBX41108, P5091, FT671 and P22077 that have been reported to inhibit USP7 in cells.^23^, ^27^, ^28^, ^29^ In recent years, several groups reported the structures of USP7 in complex with small‐molecule inhibitors^23^, ^30^ and these structures give guidance to further develop new inhibitors. The P22077 covalently modifies the catalytic cysteine of USP7 and induces a conformational switch in the enzyme associated with active site rearrangement.^31^ This makes P22077 well‐characterized tool compounds for exploring the USP7 function.^31^ In melanoma, P22077 induced an excessive ROS, which further leads to DNA damage. The excessive amounts of ROS enhance cellular oxidative stress and directly activate ATM and ATR for the DNA damage response pathway.^32^ Previous studies have shown that USP7 acts as an important regulator for DNA damage response, and inhibition or knockdown of USP7 leads to increased DNA damage via destabilization of several regulators of DNA damage such as RAD18 (replication‐associated repair), CSB (nucleotide excision repair) and ALKBH2/3 (alkylation repair).^33^, ^34^, ^35^ Moreover, USP7 also induced unfolded protein accumulation causing ER stress in cancer cells, which leads to oxidative stress to induce DNA damage. On the other hand, we also found that P22077 led inhibition of invasion and migration in vitro and in vivo in melanoma. Several substrates contributed to invasion and migration regulated by USP7, such as EZH2 in prostate cancer, and LSD1 in glioblastoma.^36^, ^37^ On the contrary, the role of ROS in triggering signalling pathways, such as activation of MAPK, ERK, JNK and p‐38 MAPK, via growth factor‐mediated stimulation of receptor tyrosine kinases (RTKs) for cell migration and invasion has been well established.^38^ Those studies indicated USP7 as a multifaceted regulator of tumorigenesis that may act through different mechanisms at the same time.

Collectively, this study demonstrated USP7 as a possible oncogenic molecule for melanoma progression, and inhibition of USP7 might be a potential strategy for the suppression of melanoma growth. Importantly, USP7 inhibitor P22077 possess antimelanoma activities in vitro and in vivo, induces apoptosis and cell cycle arrest by DNA damage and markedly impairs melanoma cell migration and invasion, indicating a prospective value of the application of P22077 as a promising novel effective strategy for melanoma therapy.

## CONFLICT OF INTEREST

The authors declared that they have no competing interests.

## AUTHOR CONTRIBUTIONS

**Minmin Xiang:** Conceptualization (lead); Data curation (lead); Formal analysis (supporting). **Long Liang:** Conceptualization (equal); Data curation (equal); Formal analysis (equal); Funding acquisition (equal). **Xinwei Kuang:** Investigation (equal); Methodology (equal). **Zuozhong Xie:** Investigation (equal); Methodology (equal). **Jing Liu:** Supervision (equal). **Shuang Zhao:** Methodology (equal). **Juan Su:** Formal analysis (equal). **Xiang Chen:** Funding acquisition (equal); Investigation (equal). **Hong Liu:** Investigation (equal); Methodology (equal); Project administration (equal).

## Supporting information

Figure S1

Figure S2

Figure S3

## Data Availability

The data sets used and/or analysed during the current study are available from the corresponding author on reasonable request.

## References

[1] Shain AH , Bastian BC . From melanocytes to melanomas. Nat Rev Cancer. 2016;16(6):345‐358. 27125352 10.1038/nrc.2016.37

[2] Ferlay J , Soerjomataram I , Dikshit R , et al. Cancer incidence and mortality worldwide: sources, methods and major patterns in GLOBOCAN 2012. Int J Cancer. 2015;136(5):E359‐E386. 25220842 10.1002/ijc.29210

[3] Ali Z , Yousaf N , Larkin J . Melanoma epidemiology, biology and prognosis. EJC Suppl. 2013;11(2):81‐91. 26217116 10.1016/j.ejcsup.2013.07.012 PMC4041476

[4] Pavri SN , Clune J , Ariyan S , Narayan D . Malignant melanoma: beyond the basics. Plast Reconstr Surg. 2016;138(2):330e‐e340. 10.1097/PRS.0000000000002367 27465194

[5] Damsky WE , Theodosakis N , Bosenberg M . Melanoma metastasis: new concepts and evolving paradigms. Oncogene. 2014;33(19):2413‐2422. 23728340 10.1038/onc.2013.194

[6] Nicholas S , Mathios D , Jackson C , Lim M . Metastatic melanoma to the brain: surgery and radiation is still the standard of care. Curr Treat Options Oncol. 2013;14(2):264‐279. 23504304 10.1007/s11864-013-0228-6

[7] Rishi A , Yu HM . Current treatment of melanoma brain metastasis. Curr Treat Options Oncol. 2020;21(6):45. 32350685 10.1007/s11864-020-00733-z

[8] Mishra H , Mishra PK , Ekielski A , Jaggi M , Iqbal Z , Talegaonkar S . Melanoma treatment: from conventional to nanotechnology. J Cancer Res Clin Oncol. 2018;144(12):2283‐2302. 30094536 10.1007/s00432-018-2726-1 PMC11813321

[9] Swatek KN , Komander D . Ubiquitin modifications. Cell Res. 2016;26(4):399‐422. 27012465 10.1038/cr.2016.39 PMC4822133

[10] Kim RQ , Sixma TK . Regulation of USP7: a high incidence of E3 complexes. J Mol Biol. 2017;429(22):3395‐3408. 28591556 10.1016/j.jmb.2017.05.028

[11] Zhou J , Wang J , Chen C , Yuan H , Wen X , Sun H . USP7: target validation and drug discovery for cancer therapy. Med Chem. 2018;14(1):3‐18. 29065837 10.2174/1573406413666171020115539

[12] Cai JB , Shi GM , Dong ZR , et al. Ubiquitin‐specific protease 7 accelerates p14(ARF) degradation by deubiquitinating thyroid hormone receptor‐interacting protein 12 and promotes hepatocellular carcinoma progression. Hepatology. 2015;61(5):1603‐1614. 25557975 10.1002/hep.27682

[13] Nicholson B , Suresh KK . The multifaceted roles of USP7: new therapeutic opportunities. Cell Biochem Biophys. 2011;60(1–2):61‐68. 21468693 10.1007/s12013-011-9185-5

[14] Altun M , Kramer HB , Willems LI , et al. Activity‐based chemical proteomics accelerates inhibitor development for deubiquitylating enzymes. Chem Biol. 2011;18(11):1401‐1412. 22118674 10.1016/j.chembiol.2011.08.018

[15] Fan YH , Cheng J , Vasudevan SA , et al. USP7 inhibitor P22077 inhibits neuroblastoma growth via inducing p53‐mediated apoptosis. Cell Death Dis. 2013;4:e867. 24136231 10.1038/cddis.2013.400 PMC3920959

[16] Li J , Han Y , Zhang H , et al. The m6A demethylase FTO promotes the growth of lung cancer cells by regulating the m6A level of USP7 mRNA. Biochem Biophys Res Commun. 2019;512(3):479‐485. 30905413 10.1016/j.bbrc.2019.03.093

[17] Turley EA , Veiseh M , Radisky DC , Bissell MJ . Mechanisms of disease: epithelial‐mesenchymal transition–does cellular plasticity fuel neoplastic progression? Nat Clin Pract Oncol. 2008;5(5):280‐290. 18349857 10.1038/ncponc1089 PMC2846172

[18] Schadendorf D , van Akkooi ACJ , Berking C , et al. Melanoma. Lancet. 2018;392(10151):971‐984. 30238891 10.1016/S0140-6736(18)31559-9

[19] Peng Y , Liu Y , Gao Y , et al. USP7 is a novel Deubiquitinase sustaining PLK1 protein stability and regulating chromosome alignment in mitosis. J Exp Clin Cancer Res. 2019;38(1):468. 31730000 10.1186/s13046-019-1457-8 PMC6858727

[20] Georges A , Marcon E , Greenblatt J , Frappier L . Identification and characterization of USP7 targets in cancer cells. Sci Rep. 2018;8(1):15833. 30367141 10.1038/s41598-018-34197-x PMC6203733

[21] Wang M , Zhang Y , Wang T , et al. The USP7 inhibitor P5091 induces cell death in ovarian cancers with different P53 status. Cell Physiol Biochem. 2017;43(5):1755‐1766. 29049989 10.1159/000484062

[22] An T , Gong Y , Li X , et al. USP7 inhibitor P5091 inhibits Wnt signaling and colorectal tumor growth. Biochem Pharmacol. 2017;131:29‐39. 28216017 10.1016/j.bcp.2017.02.011

[23] Kategaya L , Di Lello P , Rougé L , et al. USP7 small‐molecule inhibitors interfere with ubiquitin binding. Nature. 2017;550(7677):534‐538. 29045385 10.1038/nature24006

[24] Carra G , Panuzzo C , Torti D , et al. Therapeutic inhibition of USP7‐PTEN network in chronic lymphocytic leukemia: a strategy to overcome TP53 mutated/deleted clones. Oncotarget. 2017;8(22):35508‐35522. 28418900 10.18632/oncotarget.16348 PMC5482594

[25] Wang Q , Ma S , Song N , et al. Stabilization of histone demethylase PHF8 by USP7 promotes breast carcinogenesis. J Clin Invest. 2016;126(6):2205‐2220. 27183383 10.1172/JCI85747 PMC4887182

[26] Tavana O , Li D , Dai C , et al. HAUSP deubiquitinates and stabilizes N‐Myc in neuroblastoma. Nat Med. 2016;22(10):1180‐1186. 27618649 10.1038/nm.4180 PMC5091299

[27] Chauhan D , Tian Z , Nicholson B , et al. A small molecule inhibitor of ubiquitin‐specific protease‐7 induces apoptosis in multiple myeloma cells and overcomes bortezomib resistance. Cancer Cell. 2012;22(3):345‐358. 22975377 10.1016/j.ccr.2012.08.007 PMC3478134

[28] Reverdy C , Conrath S , Lopez R , et al. Discovery of specific inhibitors of human USP7/HAUSP deubiquitinating enzyme. Chem Biol. 2012;19(4):467‐477. 22520753 10.1016/j.chembiol.2012.02.007

[29] Zhang Y , Zhou L , Rouge L , et al. Conformational stabilization of ubiquitin yields potent and selective inhibitors of USP7. Nat Chem Biol. 2013;9(1):51‐58. 23178935 10.1038/nchembio.1134

[30] Turnbull AP , Ioannidis S , Krajewski WW , et al. Molecular basis of USP7 inhibition by selective small‐molecule inhibitors. Nature. 2017;550(7677):481‐486. 29045389 10.1038/nature24451 PMC6029662

[31] Pozhidaeva A , Valles G , Wang F , et al. USP7‐specific inhibitors target and modify the enzyme's active site via distinct chemical mechanisms. Cell Chem Biol. 2017;24(12):1501‐1512.e5. 29056420 10.1016/j.chembiol.2017.09.004

[32] Srinivas US , et al. ROS and the DNA damage response in cancer. Redox Biol. 2019;25: 101084. 10.1016/j.redox.2018.101084 PMC6859528 30612957

[33] Zlatanou A , Sabbioneda S , Miller ES , et al. USP7 is essential for maintaining Rad18 stability and DNA damage tolerance. Oncogene. 2016;35(8):965‐976. 25961918 10.1038/onc.2015.149

[34] Zhu Q , Ding N , Wei S , et al. USP7‐mediated deubiquitination differentially regulates CSB but not UVSSA upon UV radiation‐induced DNA damage. Cell Cycle. 2020;19(1):124‐141. 31775559 10.1080/15384101.2019.1695996 PMC6927731

[35] Zhao Y , Majid MC , Soll JM , Brickner JR , Dango S , Mosammaparast N . Noncanonical regulation of alkylation damage resistance by the OTUD4 deubiquitinase. EMBO J. 2015;34(12):1687‐1703. 25944111 10.15252/embj.201490497 PMC4475402

[36] Lee JE , Park CM , Kim JH . USP7 deubiquitinates and stabilizes EZH2 in prostate cancer cells. Genet Mol Biol. 2020;43(2):e20190338. 32453339 10.1590/1678-4685-GMB-2019-0338 PMC7252518

[37] Yi L , Cui Y , Xu Q , Jiang Y . Stabilization of LSD1 by deubiquitinating enzyme USP7 promotes glioblastoma cell tumorigenesis and metastasis through suppression of the p53 signaling pathway. Oncol Rep. 2016;36(5):2935‐2945. 27632941 10.3892/or.2016.5099

[38] Rodic S , Vincent MD . Reactive oxygen species (ROS) are a key determinant of cancer's metabolic phenotype. Int J Cancer. 2018;142(3):440‐448. 28940517 10.1002/ijc.31069

