# Supplementary figures and images for: Pharmacological inhibition of USP7 suppresses growth and metastasis of melanoma cells in vitro and in vivo
Source: J Cell Mol Med. 2021 Sep 1;25(19):9228–40. doi: 10.1111/jcmm.16834 (PMC8500953; doi:10.1111/jcmm.16834)

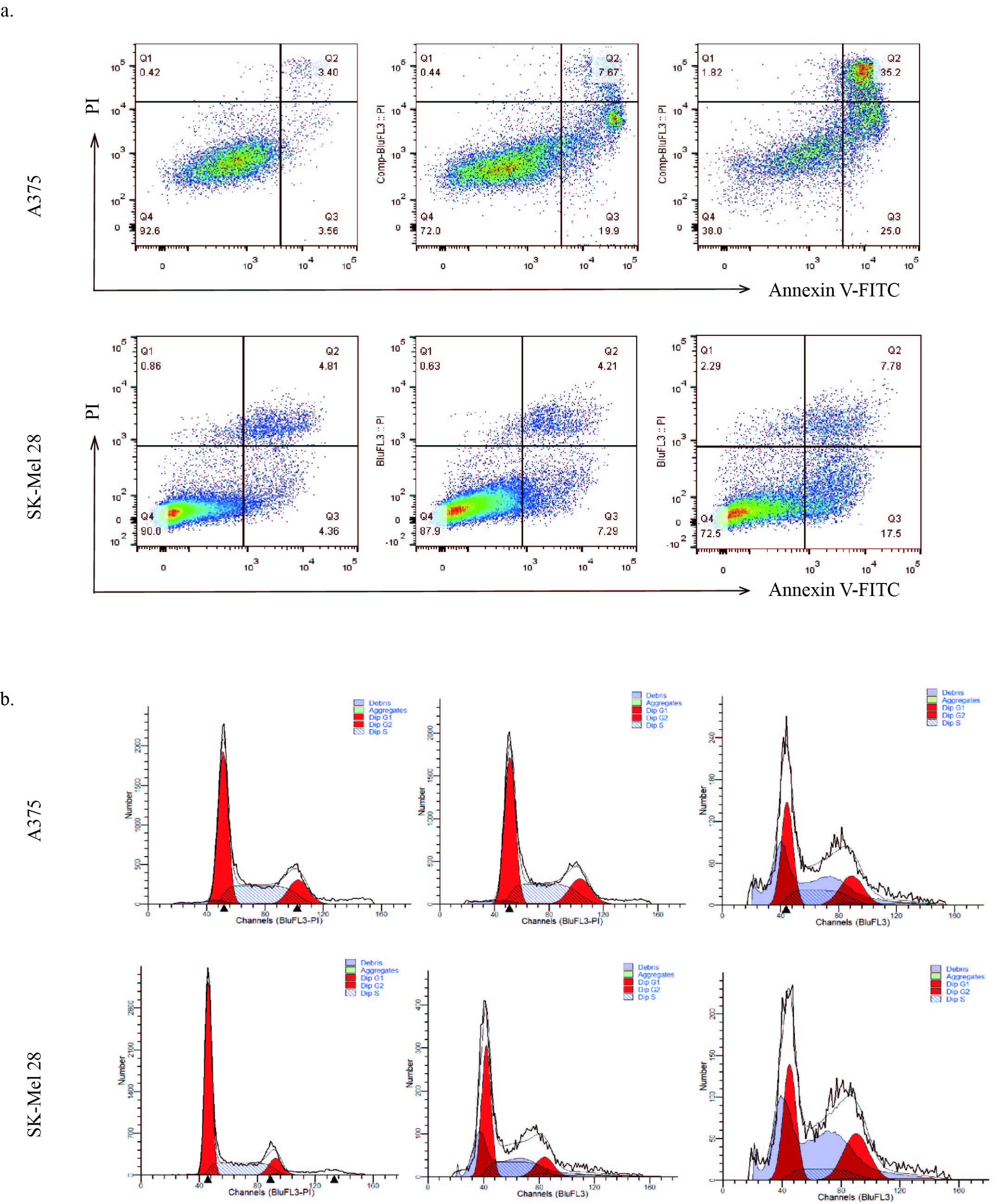

Supplement: Supplementary file 1 — Figure S1 [file JCMM-25-9228-s001.jpg]

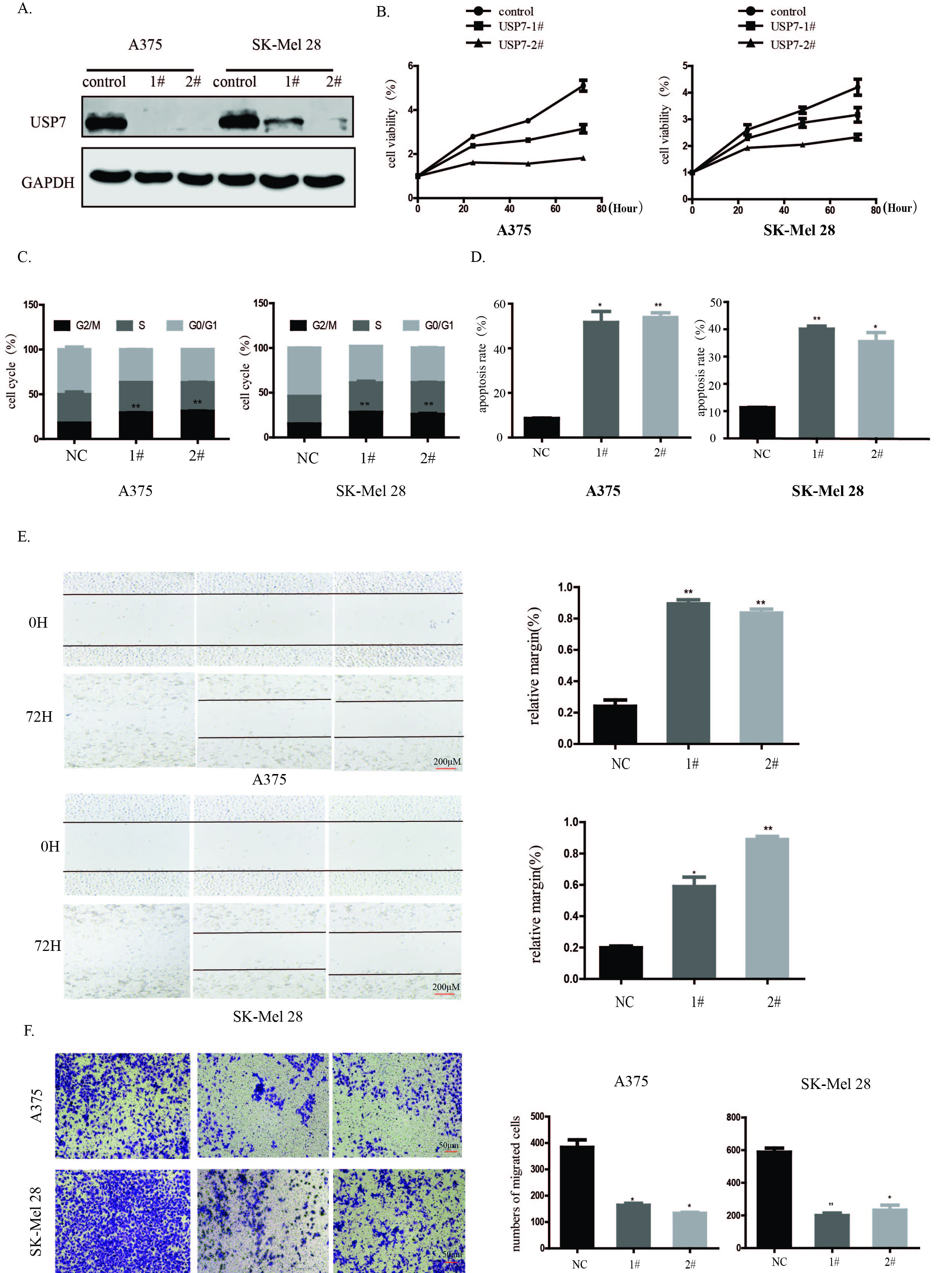

Supplement: Supplementary file 2 — Figure S2 [file JCMM-25-9228-s002.jpg]

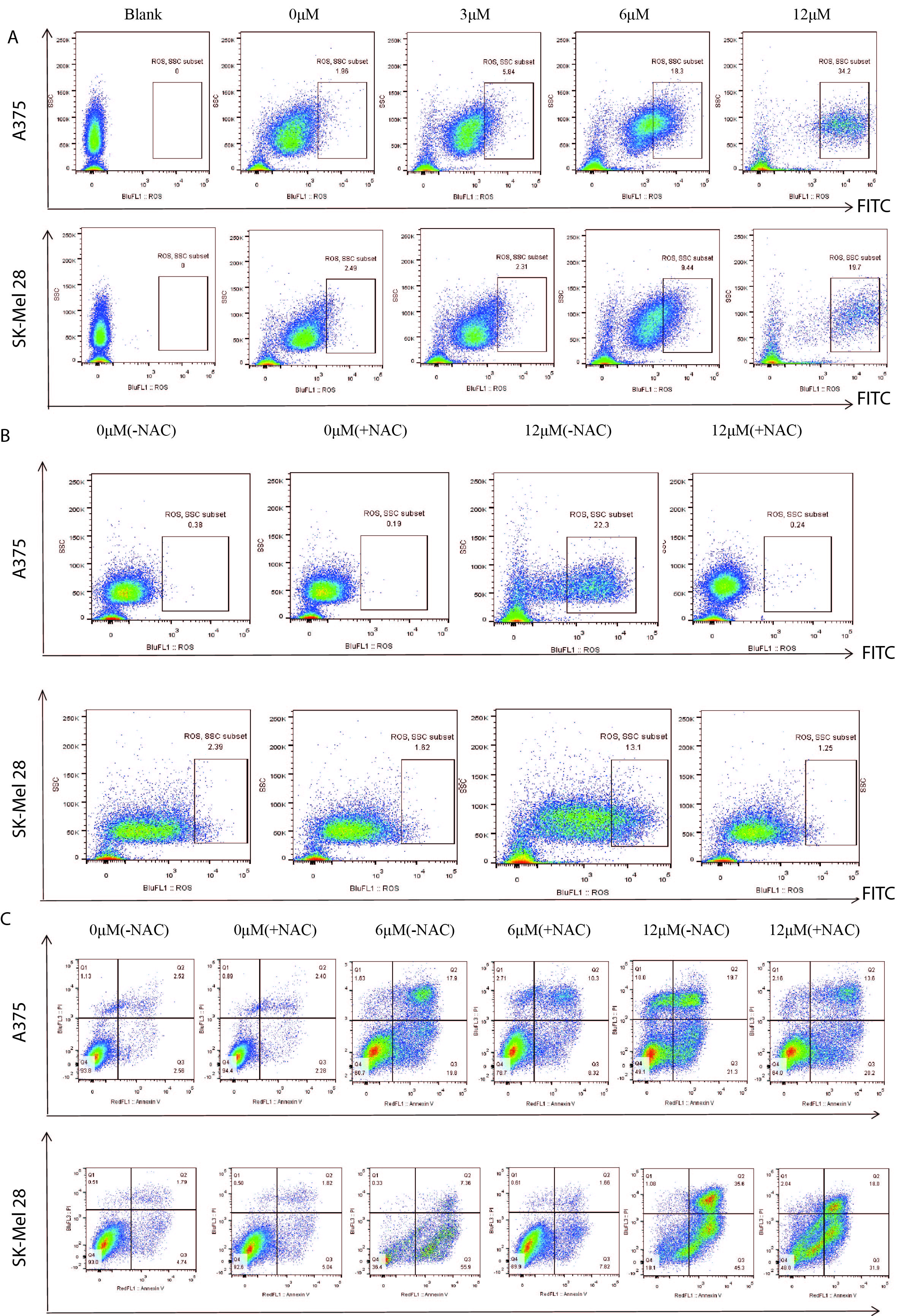

Supplement: Supplementary file 3 — Figure S3 [file JCMM-25-9228-s003.jpg]
